# Sparsely-connected autoencoder (SCA) for single cell RNAseq data mining

**DOI:** 10.1038/s41540-020-00162-6

**Published:** 2021-01-05

**Authors:** Luca Alessandri, Francesca Cordero, Marco Beccuti, Nicola Licheri, Maddalena Arigoni, Martina Olivero, Maria Flavia Di Renzo, Anna Sapino, Raffaele Calogero

**Affiliations:** 1 Department of Molecular Biotechnology and Health Science, University of Torino, Torino, Italy; 2 Department of Computer Sciences, University of Torino, Torino, Italy; 3 Department of Oncology, University of Torino, Torino, Italy; 4 Candiolo Cancer Institute-FPO, IRCCS, Candiolo (To), Candiolo, Italy; 5 Department of Medical Sciences, University of Torino, Torino, Italy

**Keywords:** Software, Software, Biomarkers

## Abstract

Single-cell RNA sequencing (scRNAseq) is an essential tool to investigate cellular heterogeneity. Thus, it would be of great interest being able to disclose biological information belonging to cell subpopulations, which can be defined by clustering analysis of scRNAseq data. In this manuscript, we report a tool that we developed for the functional mining of single cell clusters based on Sparsely-Connected Autoencoder (SCA). This tool allows uncovering hidden features associated with scRNAseq data. We implemented two new metrics, QCC (Quality Control of Cluster) and QCM (Quality Control of Model), which allow quantifying the ability of SCA to reconstruct valuable cell clusters and to evaluate the quality of the neural network achievements, respectively. Our data indicate that SCA encoded space, derived by different experimentally validated data (TF targets, miRNA targets, Kinase targets, and cancer-related immune signatures), can be used to grasp single cell cluster-specific functional features. In our implementation, SCA efficacy comes from its ability to reconstruct only specific clusters, thus indicating only those clusters where the SCA encoding space is a key element for cells aggregation. SCA analysis is implemented as module in rCASC framework and it is supported by a GUI to simplify it usage for biologists and medical personnel.

## Introduction

Single-cell RNA sequencing (scRNAseq) has emerged as essential tool to investigate cellular heterogeneity. Single cell analysis is instrumental to understand the functional differences existing among cells within a tissue. Individual cells of the same phenotype are commonly viewed as identical functional units of a tissue or organ. However, single cell sequencing results^1^ suggest the presence of a complex organization of heterogeneous cell states producing together system-level functionalities. Network analysis is a crucial tool to uncover biological and pathological mechanisms, and it is becoming an area of research for the single cell bioinformatics community. Recently, Pratapa and colleagues^2^ benchmarked 12 gene networks tools for scRNAseq, notably none of these tools exploits neural network approaches to discover functional features associated with cells’ clusters. A particular type of neural network, autoencoder, seems to be particularly suitable for the analysis of single cell data. Autoencoder is an unsupervised artificial neural network, which is designed to reduce data dimensions by learning how to ignore the noise and anomalies in the data. It first efficiently compresses and encode data and reconstructs the data back from the reduced encoded representation to produce an output that is as close as possible to the original input^3^. Autoencoder reduces data dimensions by learning how to ignore the noise in the data. Autoencoder-based approaches have been used to cluster single cell data^4^, to impute single cell data^5^, for data denoising^6^, and in batch correction^7^. Recently, Gold and co-worker^8^ have evaluated the use of autoencoders for data interpretation, implementing sparsely-connected autoencoder (SCA) to gene set analysis. SCA uses a single-layer autoencoder with sparse connections (representing known biological relationships) in order to attain a value for each gene set. SCA provides great flexibility for modeling biological phenomena^8^.

Recently, we made available to the single-cell community a framework, rCASC^9^, providing an integrated analysis environment for single-cell RNAseq. rCASC provides all the tools for sub-population discovery, which can be achieved using different clustering techniques, based on different distance metrics^9^. In this manuscript, we introduce a new rCASC module for functional annotation of cell clusters based on SCA.

## Results

### Sparsely-connected autoencoders (SCA) designed using as latent space transcription factors targets, miRNA targets, kinase targets, and cancer-related immune-signatures

**Fig. 1:**
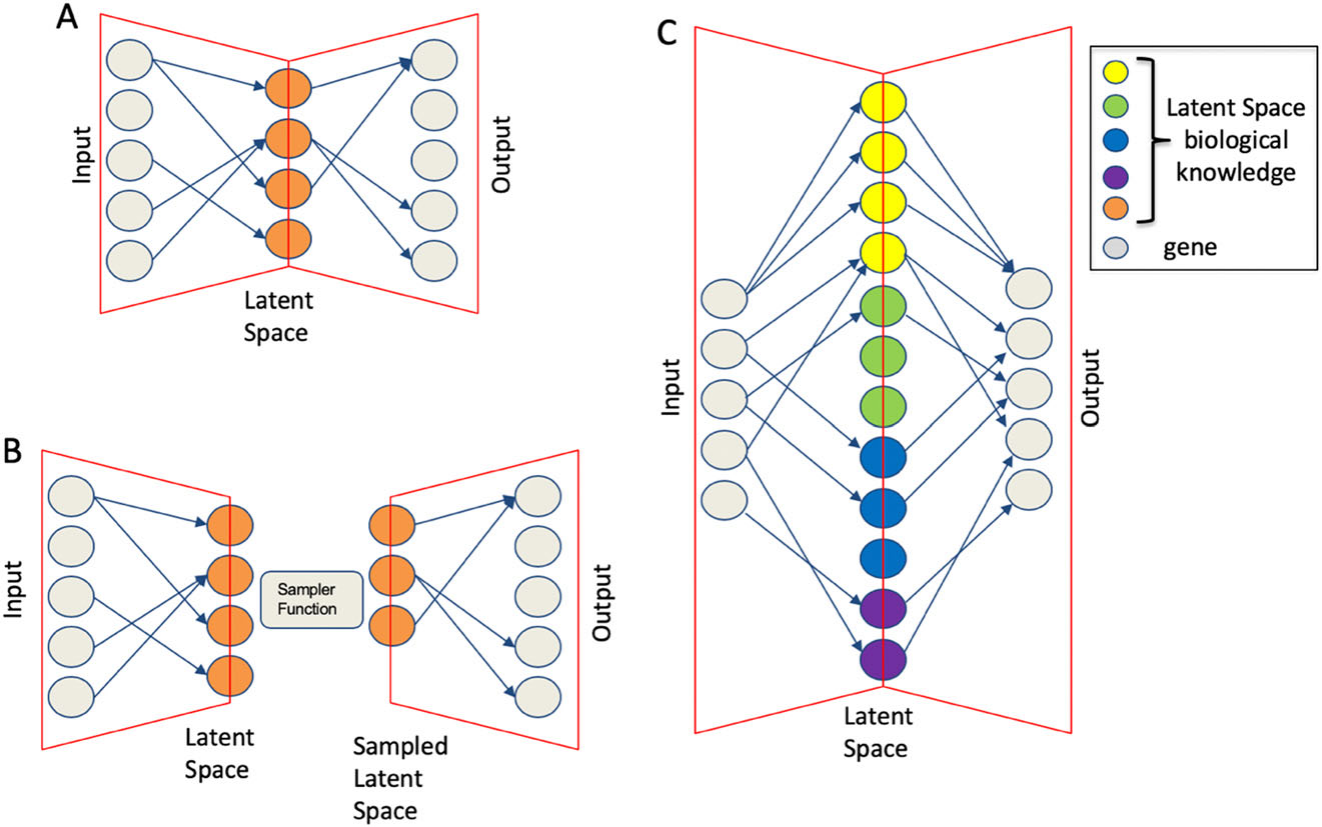
Autoencoders architecture. **a** Sparsely-connected autoencoders (SCA), (**b**) variational sparsely-connected autoencoders (vSCA), (**c**) Sparse sparsely-connected autoencoders (SSCA). Gray nodes refer to genes. Gene-level expression profiles for each gene in each cell are used as input and reconstructed as output on the basis of the latent space. The latent space is made of nodes where each node is associated with a transcription factor, a miRNA, a kinase or a functional signature or other biological knowledge. The vertices connecting input/output nodes to latent space are based on experimentally validated biological knowledge.

SCA encoding/decoding functions consisted of a single sparse layer (Fig. 1a, latent space), with connections based on known biological relationships^8,10^. Each node represented a known biological relationship, such as transcription factor (TF) targets, miRNA targets, cancer-related immune-signatures (IS), kinase (Ks) specific protein targets. SCA received inputs only from gene nodes associated with the biological relationship. With respect to the Gold paper ^8^, which used gene sets^11^, in our implementation the latent space is based only on experimentally validated data, TRRUST^12^, miRTarBase^13^, RegPhos^14^, and a manually curated cancer-based immune-signature (See “Methods” section).

The decision to use curated databases to build SCA came from the knowledge acquired over the years analyzing microarrays and bulk RNAseq data. In microarray and bulk RNAseq applications a prototypic data mining analysis is Gene Ontology (GO) enrichment, which usually nicely recapitulates the biological information hidden in a list of differentially expressed genes. However, the majority of the genes associated with GO terms were electronically inferred, thus a massive literature search, after GO enrichment analysis, is usually required to understand why a specific gene was inserted in the GO term of interest. This time-consuming literature search is instead nearly absent when curated biological knowledge is used. Specifically, we used curated databases for transcription factor targets^12^, kinase targets^14^, and experimentally validated miRNA gene targets^13^ to provide published literature information linked to the association between TFs/Kinases/miRNAs and their targets. Experimentally based annotation has the advantage of removing the noise due to the uncertainty peculiar of target prediction for miRNAs^15^. Thus, since we are querying scRNAseq datasets, which are intrinsically noisy, avoiding the introduction of extra noise due to imprecise annotation is useful to extract biological knowledge. Still, SCA can be also generated using custom defined biological knowledge, which can be based on any kind of information linked to genes, e.g., electronically inferred data like GO terms or user defined gene signatures. Independently from the data used to generate the SCA, SCA analysis must be executed multiple times on a cell dataset, previously partitioned in clusters, using any of the clustering tools implemented in rCASC: tSne+k-mean^16^, SIMLR^17^, griph^18^, scanpy^19^, and SHAR^20^. Here, we use the Cell Stability Score (CSS, for the mathematical formal description of in this metric see ref. ^9^) on SCA outputs to generate two new quality scores metrics: QCC (Quality Control of Cluster) and QCM (Quality Control of Model). QCC is generated comparing cell clusters produced by rCASC clustering, from now on called reference clusters, with the clusters generated using the latent space data after each SCA run. This metric measures the ability of the latent space in describing cells aggregations corresponding to at least part of the reference clusters. Specifically, QCC measures the frequency by which cells, belonging to a reference cluster, are found to be part of the same cluster in multiple SCA runs. QCC ranges from 0 to 1, where 0 indicates total lack of correspondence between a reference cluster and the corresponding SCA cluster over multiple runs of SCA. QCC equal to 1 indicates that cells being part of a reference cluster are detected as part of the same cluster in all runs of SCA analysis. We suggest as threshold for QCC mean a value ≥0.5, which indicates that at least in 50% of SCA runs a latent space cluster retains the structure of the cell content of the corresponding reference cluster. Instead, QCM measures cluster consistency between SCA runs. We designed this metric to evaluate the reproducibility of the model defined by SCA latent space. Specifically, if a set of biological information describing the latent space is important for the definition of a cluster, then it is expected that the majority of the SCA runs will converge to a similar solution for that cluster. Thus, comparison via QCM of random couples of clusters selected over multiple SCA runs must show a conserved cluster(s) organization. QCM ranges from 0 to 1, where 0 indicates that, in any pairs of SCA runs comparisons, there is a total lack of correspondence between the cells content of a cluster detected in a SCA run compared to the corresponding cluster in another randomly selected SCA run. Instead, QCM equal to 1 indicates that cells, being part of a SCA cluster, are always detected as part of the same cluster in any pairs of SCA runs comparisons. We suggest as threshold for QCM mean a value ≥0.5, which indicates that at least 50% of SCA runs retain the structure of the cell content in any pair of SCA runs comparison. Thus, a reference cluster explainable by SCA analysis should be characterized by both QCC and QCM ≥0.5.

As result of multiple runs of SCA, a frequency matrix is built for the latent space representations. This frequency matrix is used to detect the latent space nodes (e.g., one or more TFs), which are the most important for a cluster characterized by the mean of both QCM and QCC ≥0.5. Clusters specific signature is then detected using COMET^21^.

### Validation of SCA analysis on a PBMC derived dataset (setA)

We used a data set (setA), based on FACS purified cell types^22^, to investigate the SCA (Fig. 1a) performance. SetA was previously used to estimate the strength of CSS metric^9^. Here, we clustered setA using all the clustering tools actually implemented in rCASC: tSne+k-mean^16^, SIMLR^17^, griph^18^, Seurat^23^, scanpy^19^, and SHARP^20^. All tools but tSne+k-mean and scanpy provided very good clusters for the different cell types (see SCAtutorial section 2).

**Fig. 2:**
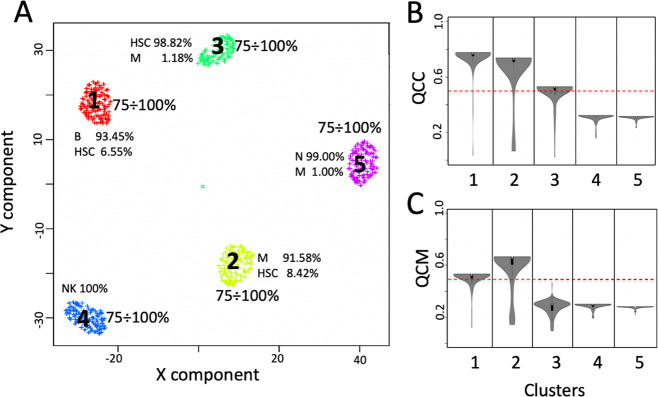
SCA analysis using a TF-based latent space. **a** Five clusters were detected analyzing setA with griph^18^ using log_10_ transformed counts table. Each cluster is made by more than 90% by one cell type. A little amount of HSC is contaminating B cells, monocytes and naïve T cells. Latent space clustering was done with SIMLR^17^. **b** QCC violin plot. The metric is an extension of CSS^9^ and it measures the ability of latent space to keep aggregated cells belonging to predefined clusters, i.e., those in panel **a**. **c** QCM violin plot, this metric is also an extension of CSS and it measures the ability of the neural network to generate consistent data over multiple SCA runs. Dashed red line indicates the defined threshold to consider the latent space information suitable to support cells’ clusters. Input counts table for SCA analysis is log10 transformed.

**Fig. 3:**
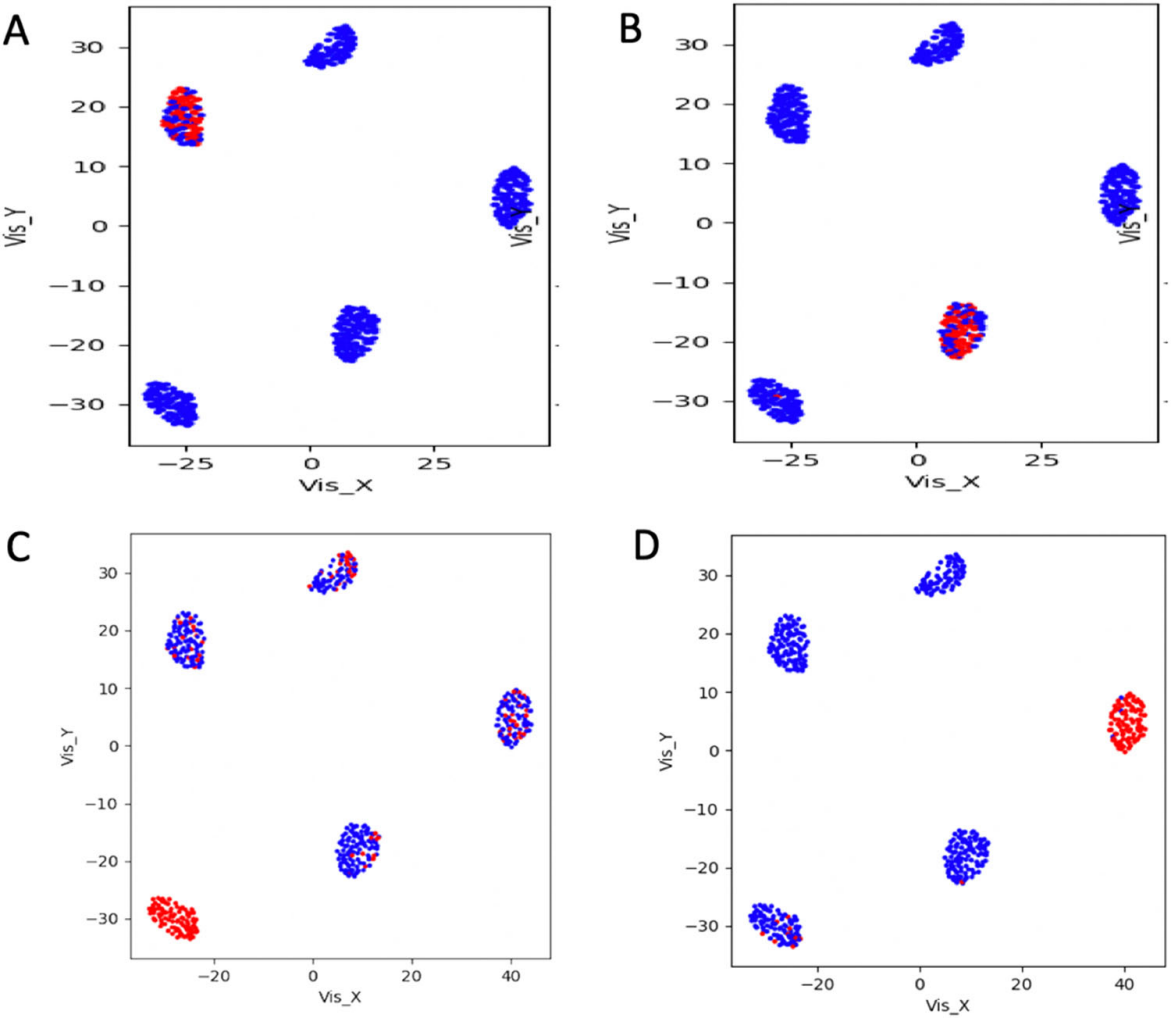
COMET analysis of latent space frequency matrix. **a** Set of 4 genes (PAX5, NFAT5, RFXANK and CHD4) characterizing cluster 1 (B cells). **b** Set of 4 genes (CEBPA, KHSRP negation, CEBPB, CREBBP) characterizing cluster 2 (Monocytes). Dots are cells, blue and red color indicate, respectively, false and true positives (for more information on this type of visualization of cluster specific markers see the COMET paper^21^. **c** Rank 1 NK signature^32^ specifically characterizing cluster 4 (NK). **d** Rank 2 Asthma KEGG negation, specifically characterizing Naïve T cells (Cluster 5).

We tested a SCA embedding a TFs-based latent space, where each latent space node was associated with a TF and arches connecting input and output nodes to each latent space node represented experimentally validated TF target genes from TRRUST database^12^. From this analysis, we observed that only cluster 1 and 2 (Fig. 2a) could be reconstructed by this type of SCA, since only these two clusters were supported by a QCC and QCM ≥0.5 (Fig. 2b, c). The matrix describing the frequency of the latent space variables was used to extract cluster specific signatures using COMET tool^21^, which is also implemented in rCASC. COMET is a computational framework for the identification of candidate marker panels consisting of one or more genes for the cell populations of interest, identified with single cell RNAseq data. The optimal maker panel definition for cluster 1 was made by four genes PAX5, NFAT5, RFXANK, and CHD4, (Table 1, Fig. 3a).

**Table 1:** Top ranked cluster-specific features detected by the analysis of the latent space using COMET software.

Latent space	Cluster	Feature 1	Feature 2	Feature 3	Feature 4	COMETsc statistics	TP	TN
SCA TF	1	PAX5	NFAT5	RFXANK	CHD4	1.45E–49	0.589	0.997
SCA TF	2	CEBPA	KHSRP negation	CEBPB	CREBBP	1.19E–46	0.561	0.997
SCA IS	4	NK signature	–	–	–	5.75E–54	1.0	0.81
SCA IS	5	ASTHMA KEGG negation	–	–	–	5.35E–84	0.970	0.972
SCA miRNA CLR	3	miR-191	–	–	–	1.01E–49	0.714	0.98
SCA miRNA RLE	3	miR-191	–	–	–	1.01E–49	0.714	0.98
SCA miRNA TMM	3	miR-132-3p	–	–	–	1.08E–49	0.714	0.98
SCA miRNA FQ	2	miR-187-3p Rank 1	–	–	–	2.85E–60	0.67	0.953
SCA miRNA SUM	2	miR-187-3p Rank 4	–	–	–	3.45E–58	0.925	0.918
SSCA	3	miR-129-2-3P	–	–	–	1.18E–49	0.742	0.98
SSCA	4	NK signature	–	–	–	6.49E–103	1.0	0.99
SSCA	5	POU2F2 negation	–	–	–	4.50E–41	0.851	0.832
vSCA TF	1	CHD4	–	–	–	9.35E–76	0.775	0.989
vSCA TF	2	CEBPA	–	–	–	6.63E–62	0.829	0.977

The four genes detected by COMET for cluster 1, which was composed mainly by B cells, recapitulated very well some of the key elements that have been shown already to be involved in development and differentiation of this cell type. The transcription factor PAX5 is essential for the commitment of lymphoid progenitors to B-lymphocyte lineage^24^. PAX5 fulfills a dual role by repressing B lineage ‘inappropriate’ genes and simultaneously activating B lineage-specific genes^24^. NFAT5 is important for optimal antibody productivity^25^. CHD4 is critical for early B cell development^26^ and RFXANK is involved in activation of MHC-II genes, which in turn MHC-II molecules are largely restricted to thymic epithelial cells and professional antigen-presenting cells, including dendritic cells, macrophages, and B cells. Moreover, for cluster 2, which was mainly composed by monocytes, the best maker panel was made of four genes, CEBPA, KHSRP (lack of expression), CEBPB, CREBBP (Table 1, Fig. 3b), which have been shown already to be strongly involved in monocyte functionalities^27^. The transcription factor CCAAT/enhancer-binding protein β (CEBPB) is highly expressed in monocytes/macrophages and is a critical factor for Ly6C-monocyte survival^28^. The downregulated expression of the KH-Type Splicing Regulatory Protein (KSRP) during monocytopoiesis and its upregulated expression during granulopoiesis suggested that KSRP has divergent roles during monocytic and granulocytic differentiation^29^. CREB is involved in anti-apoptotic survival signaling in monocytes and macrophages^30^ and CREBBP specifically binds to the active phosphorylated form of CREB^31^. A SCA analysis based on a latent space made of manually curated cancer-immune-signatures (IS, SCAtutorial, section 3) was also performed on setA reference clusters. This SCA analysis showed, for cluster 4 (Natural Killer cells) and cluster 5 (Naïve T cells), QCM/QCC values greater than 0.5 for the majority of the cells (see SCAtutorial, section 3). Analyzing the IS-based latent space frequency table with COMET, we identified one feature of the immune-signature derived from Nirmal’s paper^32^, which was characteristic of NK (Table 1, Fig. 3c) and a KEGG immune-signature, which expression absence was specific for Naïve T cells cluster (Table 1, Fig. 3d). SCA analysis on setA was also done using a latent space based on kinase targets, but we cannot find any robust association with reference clusters (see https://figshare.com/articles/dataset/Figure_3_from_manuscript_Sparsely-Connected_Autoencoder_SCA_for_single_cell_RNAseq_data_mining/12866657).

### Investigating the effect of normalization on SCA latent space frequency matrix on QCC/QCM scores

**Fig. 4:**
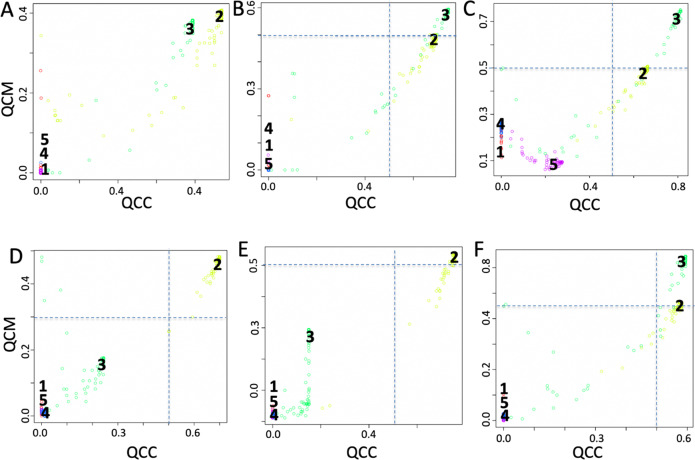
QCM/QCC plots using different normalizations for the SCA input counts table. **a** Log10 transformed, (**b**) Centered log-ratio normalization (CLR), (**c**) relative log-expression (RLE), (**d**) full-quantile normalization (FQ), (**e**) sum scaling normalization (SUM), (**f**) weighted trimmed mean of M-values (TMM).

SCA analysis based on validated target genes for miRNAs (Fig. 4a) showed that clusters 2 (Monocytes) and 3 (Hematopoietic Stem Cells) had a potentially interesting trend, although they were not supported by a QCM and a QCC ≥ 0.5. Consequently, we investigated the effect of various normalization procedures of the SCA input counts table on the modeled results, to see if normalization of SCA input data could help in improving QCM and a QCC scores. In Fig. 4, it is shown how normalization affected both QCM and QCC scores for clusters closed to the suggested significant threshold (0.5), i.e., cluster 3 (Fig. 4b, c, f) cluster 2 (Fig. 4d, e). Low quality clusters, i.e., those lacking robust latent space information, were minimally affected by normalization, i.e., clusters 1, 4 and 5. This observation suggested that it is important to assess the effect of different normalization procedures on SCA input data, specifically if SCA clusters show QCM and QCC scores near to the significant threshold (0.5) suggested for those metrics. Analyzing the latent space of miRNAs frequency table with COMET we identified (Table 1) miR-191 as top marker for cluster 3 (HSC) and ranked 1, 5, and 205 markers using CLR, RLE, and TMM normalizations, respectively. miR-191 has been already associated with the appearance of stem cell-like phenotype in liver epithelial cells^33^. Using TMM normalization for cluster 3, rank 1 marker was miR-132-3p, which has been linked to HSC maintenance^34^. miR-187-3p was detected as rank 1 marker for cluster 2 (M) with FQ normalization and as rank 4 marker in SUM normalization, respectively. miR-187 has been demonstrated to play a central role in the physiological regulation of IL-10-driven anti-inflammatory responses in TLR4-stimulated monocytes^35^. It was notable that different normalizations retained a certain amount of consistency in the top ranked markers.

### Validation of variational sparsely connected autoencoders (vSCA) analysis on a PBMC derived dataset (setA)

A variational autoencoder (VAE) consists of an encoder, a decoder, and a loss function. VAEs have one fundamentally unique property that separates them from other autoencoders: their latent spaces are, by design, continuous, allowing easy random sampling and interpolation. We applied the concept of VAE to SCA (vSCA, Fig. 1b). We tested vSCA based on TF-targets using setA. From the point of view of the QCM/QCC of clusters vSCA results were nearly superimposable to those of a TF-based SCA (see SCAtutorial, section 4). The analysis of COMET for cluster 1 and cluster 2 detected as the best markers, respectively, CHD4 and CEBPA, which were part of the 4 genes SCA signature for cluster 1 and 2 (Table 1). Taken together, these observations indicated that the vSCA, although more complex, did not provide any specific improvement with respect to a simple SCA.

### Validation of sparse sparsely connected autoencoders (SSCA) analysis on a PBMC derived dataset (setA)

**Fig. 5:**
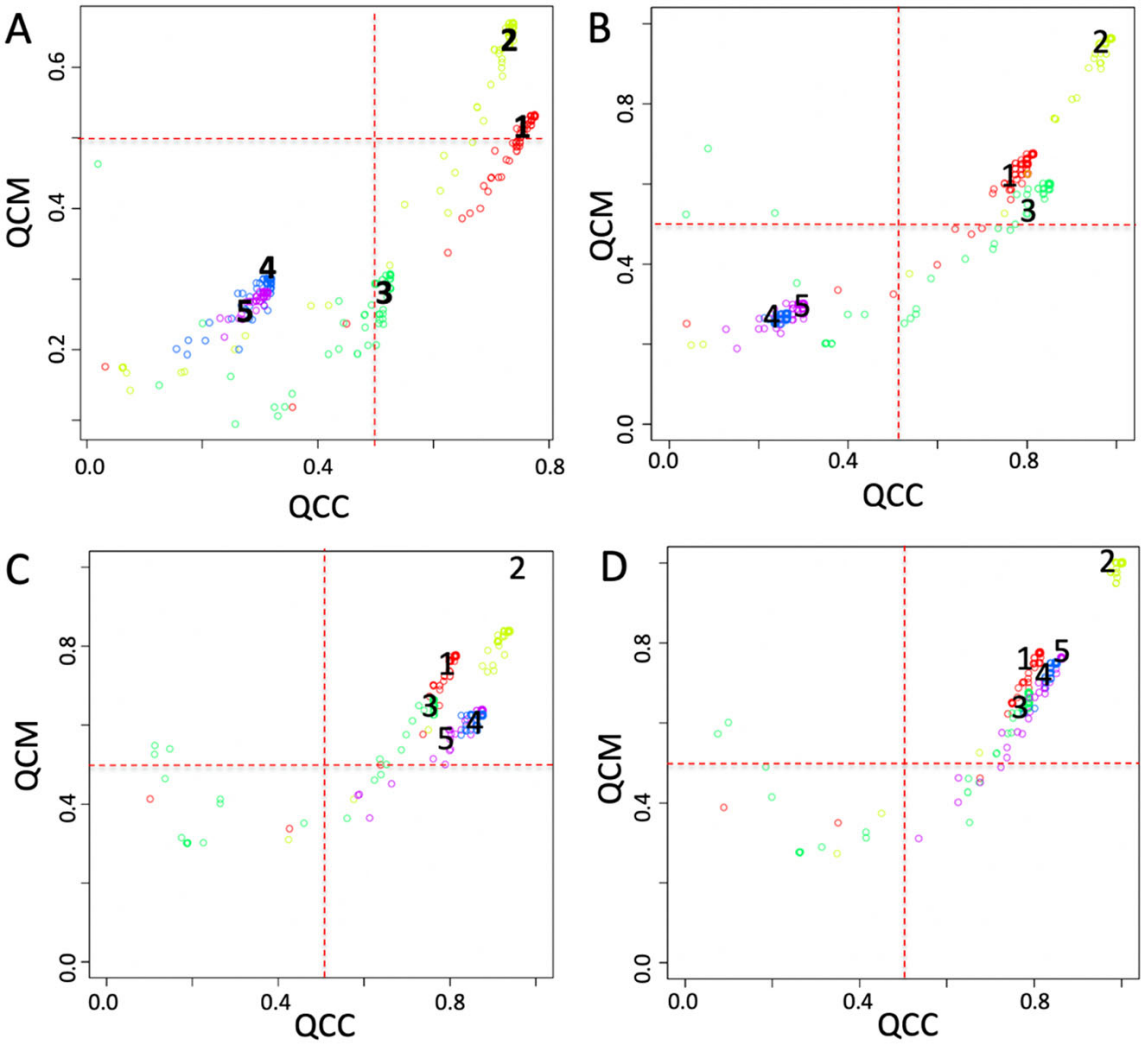
QCM/QCC plots for SSCA. **a** TF latent space. **b** TF + miRNA latent space, (**c**) TF + miRNA + IS latent space, (**d**) TF + miRNA + IS + Kinase latent space. Input counts table for SCA is log10 transformed.

Sparse autoencoder may include more hidden units than inputs, although only a small number of the hidden units are allowed to be active at once. This sparsity can improve classification performance. Usually, the sparsity is possible thanks to combinations of activation functions, sampling steps and different kinds of penalties^21^. In our implementation sparsity was generated combining TF, miRNA, IS, and kinase SCA latent spaces (Fig. 1c). The analysis of the setA using a latent space embedding integrated biological knowledge, i.e. TF + miRNA (Fig. 5b); TF + miRNA + IS (Fig. 5c); TF + miRNA + IS + Kinase (Fig. 5d), showed a notable improvement in the overall QCM/QCC scores with respect to the analysis done using each specific knowledge group alone. Furthermore, the combined latent space reduced the computing time with respect to the time needed for the independent analysis of each individual latent space.

The addition of miRNA targets to TF latent space repositioned cluster 3 (HSC) in the significant area, i.e. mean of both QCM and QCC ≥0.5, which could not be possible using only miRNA targets as latent space (Fig. 4a). For cluster 3 the best marker was miR-129-2-3P. Interestingly, miR-132, detected as top ranked for cluster 3 upon TMM normalization (Fig. 4f), together with miR-129 from SSCA analysis, were distinct faces of the same coin, since miR-132 has been already shown to be linked to HSC maintenance^34^ and miR-129 was found to be associated to self-renewal and lineage differentiation of stem cells^36^.

The addition of the IS to TF + miRNA latent space relocated in the significant area cluster 4 (NK cells) and 5 (naïve T cells) (Fig. 5c), as in the case of IS-based latent space alone (see SCAtutorial, section 3). For cluster 4, the first top ranked item was NK signature^37^. Instead, for cluster 5 (Naïve T cells) the best marker was of the expression absence of transcription factor POU2F2, which is expressed only in activated T cells^38^. ASTHMA KEGG pathway lacks of expression was also detected using IS-based SCA alone (Fig. 3d). Notably, the Kinase based latent space alone was not able to bring any cluster in the significant area (see SetA_Kinome dataset at https://figshare.com/projects/Sparsely-Connected_Autoencoder_SCA_for_single_cell_RNAseq_data_mining/88247). However, when Kinases are added to TF + miRNA + IS latent space (Fig. 5c), the QCM and QCC scores for cluster 4 and 5 improved slightly, but kinases were not present in the top ranked genes for cluster 4 and 5.

### Application of SCA analysis on spatially resolved transcriptomics of breast cancer histological section

As example for the useful application of SCA analysis, we analyzed a breast cancer histological section available as part of the demo data proposed for visium, i.e., spatially resolved transcriptomics (10XGenomics, USA)^35^. Spatially resolved transcriptomics provides the sequencing of up to 5000 spots (55 µm ∅) of a histological tissue (6.5 × 6.5 mm) section embedded in OTC. This technology does not guarantee single cell sequencing, since in each spot, on the basis of the cells size and density, there could be between 1 and 30 cells.

**Fig. 6:**
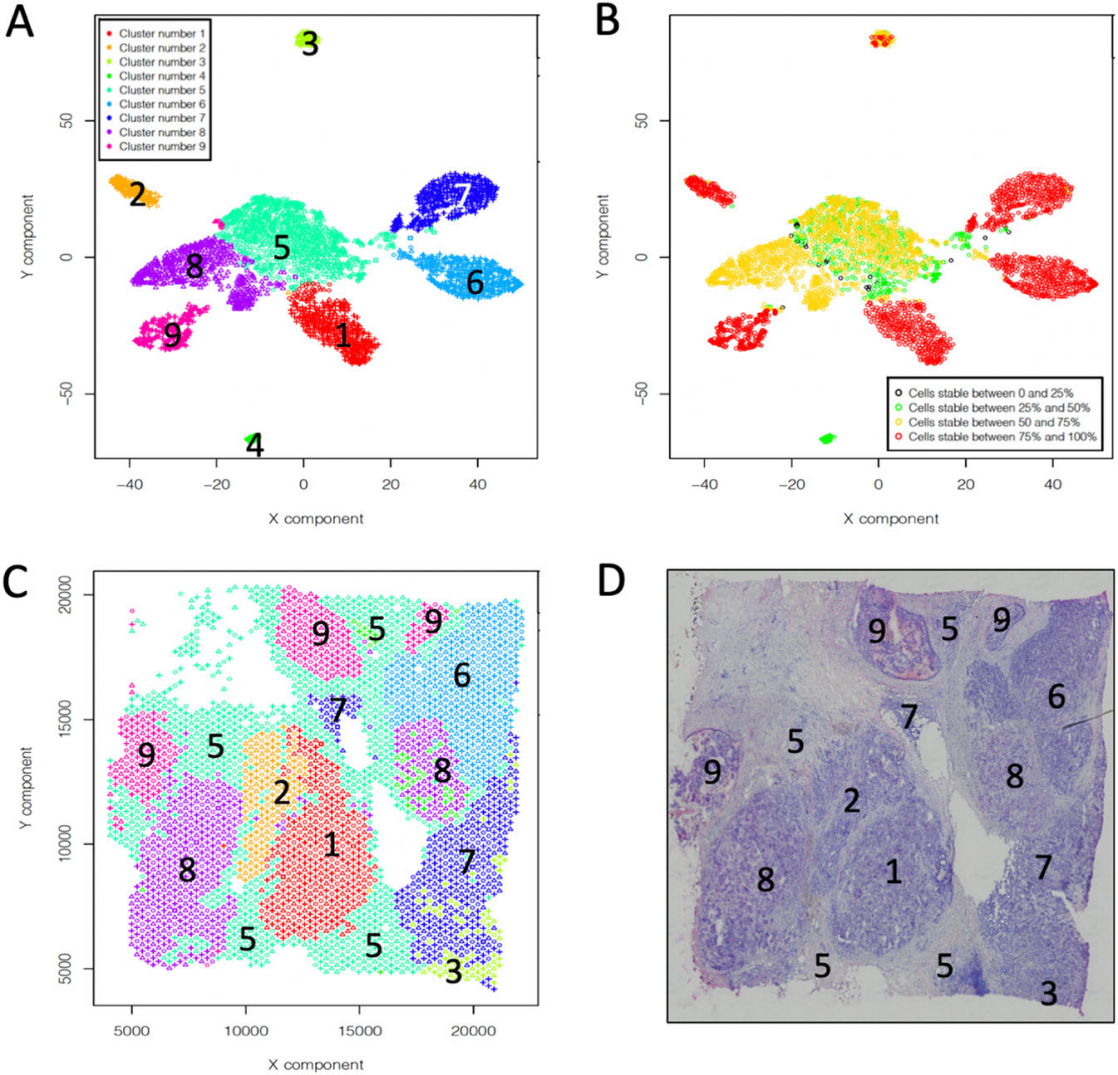
Analysis of human breast cancer (Block A Section 1), from 10XGenomics Visium Spatial Gene Expression 1.0.0. demonstration samples. **a** SIMLR partitioning in 9 clusters. **b** Cell stability score plot for SIMLR clusters in A. **c** SIMLR clusters location in the tissue section. **d** Hematoxylin and eosin image.

We obtained the best clustering organization of expression data using SIMLR (Fig. 6a), which generated a partition made of 9 clusters, where 6 clusters (1, 2, 3, 6, 7, 8) showed very high CSS (Fig. 6b), while other two clusters (5 and 8) showed an intermediate but still significant CSS (Fig. 6b). Clusters were then localized on the histological session (Fig. 6c, d). Despite the low magnification of histological picture (Fig. 6d) obtained from the frozen section, the pathologists were able to assign cluster 5 to areas predominantly corresponding to tumor stroma; cluster 9 to ductal carcinoma in situ with micropapillary features; clusters 1 and 8 corresponded to roundish areas annotated as invasive carcinoma, showing the same dyeability and possibly histological similarity (micropapillae) with areas associated with cluster 9. Clusters 6 and 7 were allocated to invasive carcinomas with comparable features at low magnification (smaller cell clusters infiltrating the stroma). Cluster 3 and 4 could not be classified by the pathologists, because of the limited number of cells.

**Fig. 7:**
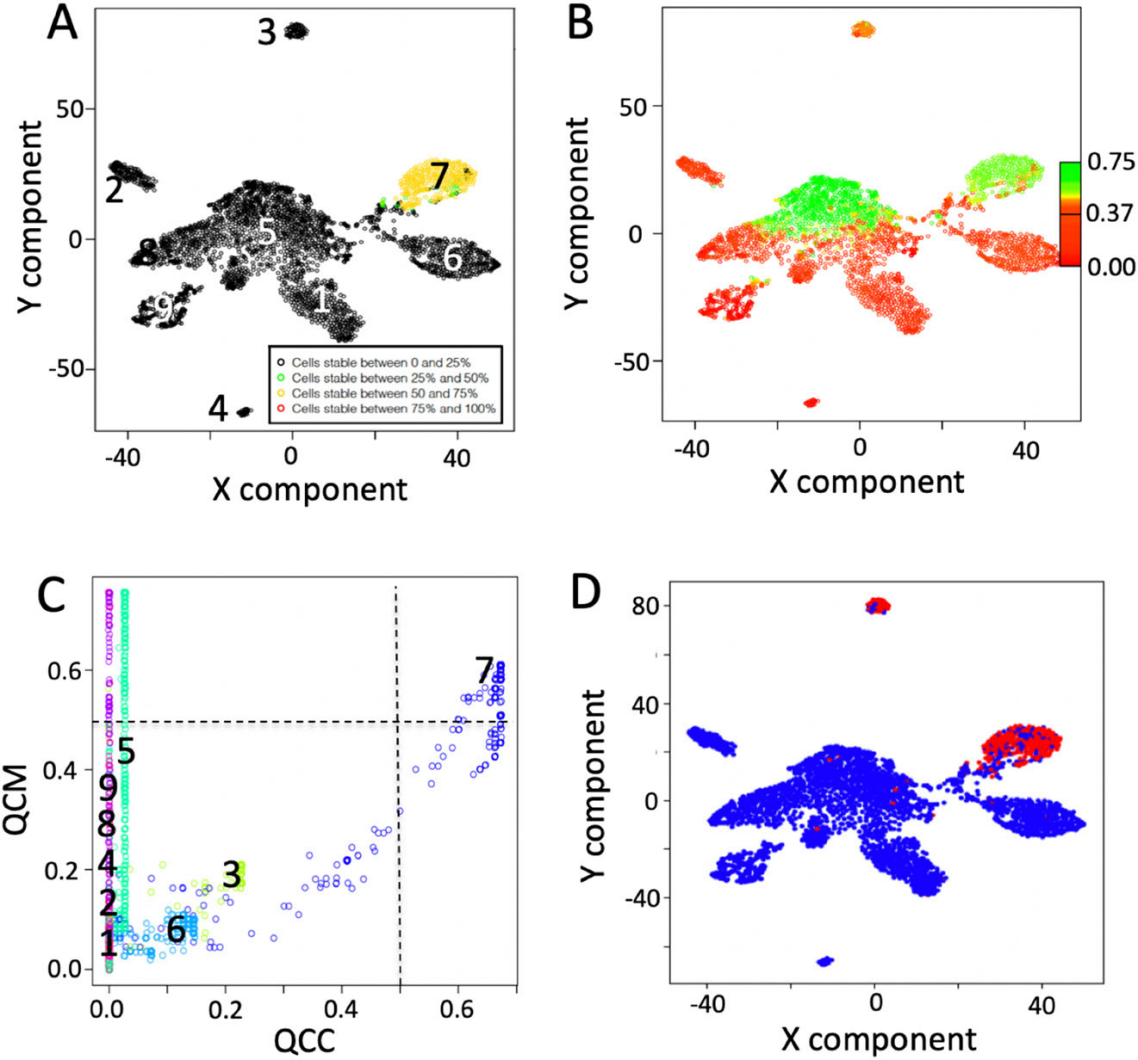
Information contents extracted by SCA analysis using a TF-based latent space. **a** QCC. **b** QCM. **c** QCM/QCC plot, where only cluster 7 shows, for the majority of the cells, both QCC and QCM greater than 0.5. **d** COMET analysis of SCA latent space. SOX5 was detected as first top ranked gene specific for cluster 7, using as input for COMET the latent space frequency table. Input counts table for SCA analysis is made by raw counts.

We tested the ability of SCA to associate TFs with the detected clusters and only cluster 7 could be described by SCA analysis (Fig. 7). COMET analysis of the latent space frequency table provided the detection of SOX5 (COMETsc statistics = 2.88E−184, TP = 0.623, and TN = 0.968) as top ranked transcription factor specific for cluster 7 (Fig. 7d). Notably, SOX5 has been recently associated with breast cancer proliferation and invasion^39^, suggesting a peculiar aggressive phenotype for the invasive carcinoma associated with cluster 7. We also tested miRNA, immune signature and kinase based SCA, but we could not find any robust association with reference clusters (see https://figshare.com/articles/dataset/Figure_6_and_7_from_manuscript_Sparsely-Connected_Autoencoder_SCA_for_single_cell_RNAseq_data_mining/12866897). These observations suggested that the knowledge present in SCA based on miRNA, immune signature and kinase targets were not sufficient to describe the complexity of tumors clusters observed in this specific dataset. At the same time the results obtained for cluster 7, using the TF-based latent space, highlighted the ability of SCA to grasp specific knowledge associated with transcription control in this experimental setting.

## Discussion

Gold and coworkers^8^ proposed SCA as a promising tool for projecting gene-level data onto gene sets. Indeed, their results suggest that SCA can be efficiently exploited in the identification of transcription factors with differential activity between conditions or cell types. Moreover, they provided some preliminary indications on how SCA can be exploited as a tool for cell classification. However, Gold and coworkers^8^ did not release any implementation of these methods, and they did not report a well-defined scoring metric to evaluate the efficacy of SCA in grabbing biological information in scRNAseq experiments. In this manuscript, a new application for SCA was introduced. Instead of using SCA to classify cell subpopulations^8^, SCA was exploited to query cell subpopulations to discover the functional features (e.g., TFs, miRNAs, Kinases, etc.) driving cell clusterization. To achieve this goal, we developed a user-friendly implementation of SCA and designed specific scoring metrics to evaluate the efficacy of SCA analysis, i.e., QCM metric that emphasizes the robustness of autoencoder-based methods measuring consistency among several SCA runs and QCC metric that empowers the strength of the autoencoder detected functional features, by measuring consistency among cell subpopulations and SCA clusters. Specifically, we investigated the ability of SCA to reproduce, completely or partially, cell clusters organization depictable from a scRNAseq experiment. Here, we show that different hidden layers, derived by experimentally validated data (TF targets, miRNA targets, Kinase targets, and cancer-related immune signatures), can be used to grasp single cell cluster-specific functional features. Then, when SCA encoding is able to reconstruct at least one of the clusters, observable aggregating cells on the basis of their full transcriptome, that means that the encoded biological knowledge is mandatory to obtain a specific aggregation of cells.

In our implementation, SCA efficacy comes from its ability to reconstruct only specific clusters, thus indicating only those clusters where the SCA encoding space is a key element of a specific cell subgroup. This is clearly demonstrated looking at the top ranked transcription factors, derived from SCA encoded space for cluster 1 and 2 of the blood-cell based dataset (setA), which were shown to be key elements for B cells and monocytes.

A very important element of our SCA implementation is the availability of metrics estimating the robustness of the SCA encoding. QCC is used to evaluate SCA coherence with respect to predefined cell clusters and QCM provides a measure for the overall SCA model quality. Furthermore, the effect of SCA input count table normalization on SCA encoding can be also estimated using QCC and QCM scores. Thus, this allows us to define the optimal condition to retrieve biological knowledge from the SCA encoded space.

Furthermore, integrating different biological knowledge in the latent space of SSCA has the advantage of reducing the computing time, since it combines different biological knowledge, i.e., TFs, miRNAs, IS, Kinases, in a unique latent space, and at the same time retains and refines the biological information that can be retrieved using independent analysis on SCA based on one biological information at a time, e.g., the SSCA signature for cluster 5 (naïve T cell) refined the definition of the cluster, since POU2F2 negation fits well with naïve T cell.

ScRNAseq although powerful has the limit of being very noisy^40^. A particularly prominent aspect of noise is dropout, i.e., scRNA-seq produces more zeros than expected and this bias is greater for poorly expressed genes^41^. Transcription factors and kinases are encoded by genes characterized by a relatively low expression in cells, thus they can be notably affected by dropout. Furthermore, nowadays it is not possible to quantify, at single cell level, miRNAs together with mRNAs. Thus, important functional networks, e.g., TF-miRNA circuits, characterizing a cell subpopulation, cannot be directly measured. In this manuscript, we show that SCA is able to grasp hidden knowledge present in cell subpopulations. Thus, SCA offers a fresh view of regulatory genes that, because of scRNAseq noise, cannot be efficiently quantified, such as transcription factors and kinases, or not detected at all, i.e., miRNAs. Furthermore, SCA based on specific signature, such as immune signature, can help in refining the annotation of cell subpopulations.

Last but not least, since SCA usage could be particularly challenging for life science and medical personnel, lacking of strong computation skill, our implementation of SCA within rCASC framework^9^ solves the above issue, because SCA is also fully accessible via GUI.

## Methods

### Datasets

Dataset setA is based on FACS purified cell types^22^. It is made of 100 cells from five cell types: B cells (B), Monocytes (M), Natural Killer (NK), Naïve T cell (N), Hematopoietic Stem Cells (HSC). This dataset was analyzed without any filtering. Data were log_10_ transformed before clustering.

Dataset HBC_BAS1 is derived from 10XGenomics spatial transcriptomics datasets resources^42^. The filtered sparse matrix from 10XGenomics repository was transformed in a dense matrix using rCASC *h5tocsv* function. Dataset was annotated using ENSEMBL Homo sapiens GRCh38.99 GTF file using the rCASC *scannobyGtf* function. After annotation, ribosomal and mitochondrial protein genes were removed together with all ENSEMBL ID not belonging to protein_coding ENSEMBL biotype. Cells with less than 250 detected genes were also removed (i.e., a gene is called detected if it is supported by at least 3 UMIs). After filtering rCASC *topx* function was used to select the 10000 most dispersed genes and from them the 5000 most expressed genes. The final matrix was made by 5000 genes and 3432 cells out of the initial 3813 cells (HBC_BAS1). Data were log_10_ transformed before clustering.

### Model coding and hyperparameter selection

This work uses sparsely-connected autoencoders^8^ (Fig. 1) to grasp cluster-specific hidden features. Autoencoders learning is based on an encoder function that projects input data onto a lower dimensional space. Then, a decoder function recovers the input data from the low-dimensional projections minimizing the reconstruction. We implemented the models in python (version 3.7) using TensorFlow package (version 2.0.0), Keras (version 2.3.1), pandas (version 0.25.3), numpy (version 1.17.4), matplotlib (version 3.1.2), sklearn (version 0.22), scipy (version 1.3.3). Optimization was done using Adam (Adaptive moment estimation) using the following parameters lr = 0.01, beta_1 = 0.9, beta_2 = 0.999, epsilon = 1e−08, decay = 0.0, loss = ‘mean_squared_error’. RELU (rectified linear unit) was used as activation function for dense layer.

The SCA input gene table could be made by raw or log_10_ transformed or normalized counts, using one of the following tools implemented in rCASC: (i) centered log-ratio normalization; (ii) relative log-expression; (iii) full-quantile normalization; (iv) sum scaling normalization; (v) weighted trimmed mean of M-values.

### SCA and SSCA latent space definition

SCA latent space is generated using as input a tab delimited text file having as first column the feature id associated with the latent space node and a second column having the input/output gene associated to the latent space node. Third column is compulsory and includes the reference from which the feature/gene relation was taken.

Experimentally validated transcription factors’ target genes and the associated transcription factor were retrieved from TRRUST v2.0^12^. Experimentally validated miRNA gene targets and their corresponding miRNA were retrieved from miRTarBase v8.0^43^. Kinases target genes were retrieved from RegPhos v2.0 database^14^. Cancer immune-signature was manually curated, retrieving genes ids from PUBMED articles related to the keyword “cancer immune signature” and genes derived from KEGG “Immune system” pathways^44^. Genes associated with KEGG pathways were manually extracted from KEGG pathways public repository^45^. SSCA latent space is made by the union of TF, miRNA, IS and Kinases data.

### QCM and QCC metrics

QCM and QCC are extensions of CSS^9^. QCC describes the cell stability of a reference cluster with respect to a cluster generated using SCA latent space information. Reference clusters are those generated using any of the clustering tools implemented in rCASC^9^. In QCC, reference clusters are compared to multiple runs SCA, where clusters are constructed using latent space information. High coherence between a reference cluster and a SCA cluster indicates that latent space is able to properly describe reference cluster organization using only the biological knowledge embedded in it. The QCC threshold for an informative latent space cluster is a value grater or equal to 0.5, i.e., in 50% of the SCA runs cells are colocalizing as in corresponding reference cluster.

QCM is instead measuring the robustness of the SCA model. Each run of the SCA the latent space starts from a random configuration, which is modeled on the basis of the information provided to the SCA, i.e., gene counts. Thus, if the SCA latent space describes properly some of the reference clusters, then those clusters should remain similar among various runs of SCA. QCM measures the reproducibility of each single cluster over a large set of randomly pairs of SCA runs. The lack of reproducibility between clusters indicates that latent space information is not relevant or not robust enough to support conserved cluster structures. The QCM threshold describing a robust model for a cluster is a value grater or equal to 0.5, i.e., in 50% of random pairs of SCA runs cells are colocalizing in the same cluster.

### COMET analysis

The cluster-specific markers detection was done using COMET^21^, which is implemented in rCASC. COMET was set to extract up to 4 features characterizing each cluster. Although COMET analyses all available clusters, marker features are investigated only for those clusters characterized by the mean of both QCM and QCC ≥0.5.

### SCA handling functions in rCASC

A full description of the SCA handling functions, available in rCASC, are described in SCAtutorial github (https://github.com/kendomaniac/SCAtutorial), which includes a vignette (https://kendomaniac.github.io/SCAtutorial/articles/SCAvignette.html) and the outputs of the exemplary analysis (https://github.com/kendomaniac/SCAtutorial/tree/master/vignettes/setA).

### Reporting summary

Further information on research design is available in the Nature Research Reporting Summary linked to this article.

## Supplementary information

Reporting summary

## Data Availability

The datasets generated and analyzed during this study are available in the figshare project “Sparsely-Connected Autoencoder (SCA) for single cell RNAseq data mining”, https://figshare.com/projects/Sparsely-Connected_Autoencoder_SCA_for_single_cell_RNAseq_data_mining/88247.
